# Anti-Inflammatory Activities of Natural Products Isolated from Soft Corals of Taiwan between 2008 and 2012

**DOI:** 10.3390/md11104083

**Published:** 2013-10-23

**Authors:** Wen-Chi Wei, Ping-Jyun Sung, Chang-Yih Duh, Bo-Wei Chen, Jyh-Horng Sheu, Ning-Sun Yang

**Affiliations:** 1 Agricultural Biotechnology Research Center, Academia Sinica, Taipei 128, Taiwan; E-Mail: jackwei@gate.sinica.edu.tw; 2 National Museum of Marine Biology & Aquarium, Pingtung 944, Taiwan; E-Mail: pjsung@nmmba.gov.tw; 3 Graduate Institute of Marine Biotechnology, National Dong Hwa University, Pingtung 944, Taiwan; 4 Department of Marine Biotechnology and Resources, National Sun Yat-sen University, Kaohsiung 804, Taiwan; E-Mails: yihduh@mail.nsysu.edu.tw (C.-Y.D.); a6152761@yahoo.com.tw (B.-W.C.); 5 Department of Medical Research, China Medical University Hospital, China Medical University, Taichung 404, Taiwan; 6 Graduate Institute of Natural Products, Kaohsiung Medical University, Kaohsiung 807, Taiwan; 7 Institute of Biotechnology, National Taiwan University, Taipei 106, Taiwan; 8 Department of Life Science, National Central University, Taoyuan 320, Taiwan

**Keywords:** soft coral, anti-inflammatory activity, iNOS, COX-2, superoxide anion, elastase

## Abstract

This review reports details on the natural products isolated from Taiwan soft corals during the period 2008–2012 focusing on their *in vitro* and/or *in vivo* anti-inflammatory activities. Chemical structures, names, and literature references are also reported. This review provides useful and specific information on potent anti-inflammatory marine metabolites for future development of immune-modulatory therapeutics.

## 1. Introduction

Marine natural products, especially those from stationary or slow moving marine organisms, are used naturally as a chemical defense to protect the organisms from dangerous predators, stressful local environments, and/or the encroachment of competitors. Due to the biological and chemical diversity of marine habitats, and the identification and greater understanding of marine secondary metabolites with unique chemical structures and biological activities, natural products from marine organisms are increasingly being considered as a major source of new therapeutics [1,2,3]. More than 20,000 novel compounds have been isolated and identified from marine organisms since the 1960s [4]. At least two current drugs and a series of anti-tumor drug candidates in preclinical or clinical trials have been developed from marine natural products [2,3,4]. The soft corals or Alcyonacea, an order of Anthozoa widely distributed in warm seawaters, have been a particular focus of attention. An abundance of unique secondary metabolites including sesquiterpenoids, diterpenoids, steroids and other chemical compounds have been isolated and identified from various species of soft corals [5,6,7]. It has been estimated that the percentage of new metabolites discovered from soft corals represents up to 22% of the total new marine natural products reported from 2010 to 2011 [5,6]. Importantly, many of the natural products discovered from soft corals have been demonstrated to exhibit a spectrum of biological activities such as anti-tumor, antiviral, antifouling and anti-inflammatory [5,6,7,8]. 

Inflammation processes often constitute an initial activation of the mammalian immune system, and the body’s normal defense or protective mechanisms in response to microbial infection or irritation or injury of tissues/organs. Increasing evidence suggests a critical link between inflammation and the chronic promotion/progression of various human diseases, including atherosclerosis, diabetes, arthritis, inflammatory bowel disease, cancer and Alzheimer. Proinflammatory enzymes, particularly the inducible nitric oxide synthase (iNOS) for nitric oxide production and cyclooxygenase (COX-2) for prostaglandin production, have been demonstrated to play central roles in the development of inflammatory diseases. In addition, it is also known that during the initial phase of acute inflammation, neutrophils are one of the first leukocyte populations to migrate towards the damaged tissue sites [9]. Neutrophils play a key role in the pathogenesis of various chronic inflammation diseases such as rheumatoid arthritis [10,11]. Activated neutrophils can secrete the superoxide anion, reactive oxygen species (ROS) and enzymes that are associated with the killing of invading pathogens [12]. Furthermore, elastase secreted by stimulated neutrophils has been recognized to play a key contribution in the demolition of tissues affected by chronic inflammatory disease [13]. Therefore, evaluation of the inhibition of iNOS and COX-2 expression, the production of superoxide anion, and the release of elastase in inflammatory cells/tissues by various natural products have been extensively employed in a spectrum of *in vitro* preliminary screening systems for lead compound or drug discovery. Recently, a number of marine biology and chemistry researchers in Taiwan (including our laboratory) have systematically screened several marine natural products isolated from soft corals for such *in vitro* anti-inflammatory activities, mainly by measuring the inhibition of iNOS, COX-2, superoxide anion or elastase in murine immune cells. Animal models were further used to evaluate the potential therapeutic activities of candidate compounds in specific disease models. This report reviews some recent representative studies and examples of marine natural products with anti-inflammatory and other related bioactivities that have been isolated from soft corals of Taiwan. Soft corals are abundant in the off-shore environment of the island of Taiwan, and have hence become a focus of local studies of marine nature products. We hope that this review will provide a useful data for the further study of marine natural products.

## 2. Results and Discussion

In the reports reviewed here, anti-inflammatory activities of natural products from the soft corals of Taiwan were generally determined *in vitro* by their inhibition of LPS-induced expression of iNOS and COX-2 in murine macrophage cells (RAW264.7) or by their inhibition of the production of superoxide anion and the release on the elastase from human neutrophils in response to FMLP/CB.

### 2.1. Sesquiterpenoids

#### 2.1.1. Triquinane-Type Sesquiterpenoids

**Figure 1:**
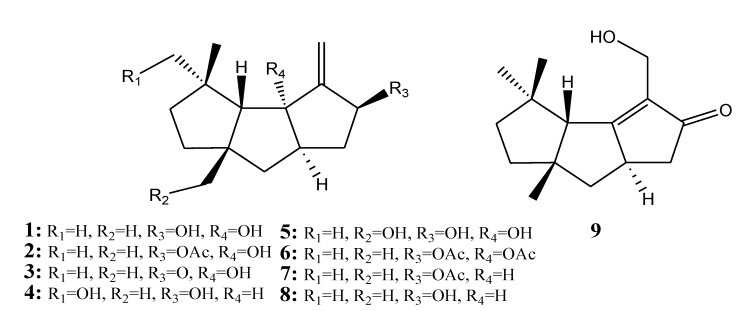
The structures of triquinane-type sesquiterpenoids (**1**–**9**).

Table 1 summarizes nine triquinane-type sesquiterpenoids (**1**–**9**) evaluated for *in vitro* anti-inflammatory activity in literature published from 2008 to 2012. The corresponding chemical structures are reported in Figure 1.

**Table 1:** Chemical constituents of triquinane-type sesquiterpenoids from soft corals of Taiwan.

No.	Name	Sources	Activities *	Reference
**1**	Δ^9(12)^-Capnellene-8β,10α-diol	*Capnella imbricata*	I,C	[14]
**2**	8α-Acetoxy-Δ^9(12)^-capnellene-10α-ol	*Capnella imbricata*	I,C	[14]
**3**	Δ^9(12)^-Capnellene-10α-ol-8-one	*Capnella imbricata*	I	[14]
**4**	Δ^9(12)^-Capnellene-8β,15-diol	*Capnella imbricata*		[14]
**5**	Δ^9(12)^-Capnellene-8β,10α,13-triol	*Capnella imbricata*		[14]
**6**	8β,10α-Diacetoxy-Δ^9(12)^-capnellene	*Capnella imbricata*		[14]
**7**	8β-Acetoxy-Δ^9(12)^-capnellene	*Capnella imbricata*		[14]
**8**	Δ^9(12)^-Capnellene-8β-ol	*Capnella imbricata*		[14]
**9**	Δ^9(12)^-Capnellene-12-ol-8-one	*Capnella imbricata*	I,C	[14]

* Inhibition of iNOS (I) and COX-2 (C).

#### 2.1.2. Nardosinane-Type Sesquiterpenoids

**Figure 2:**
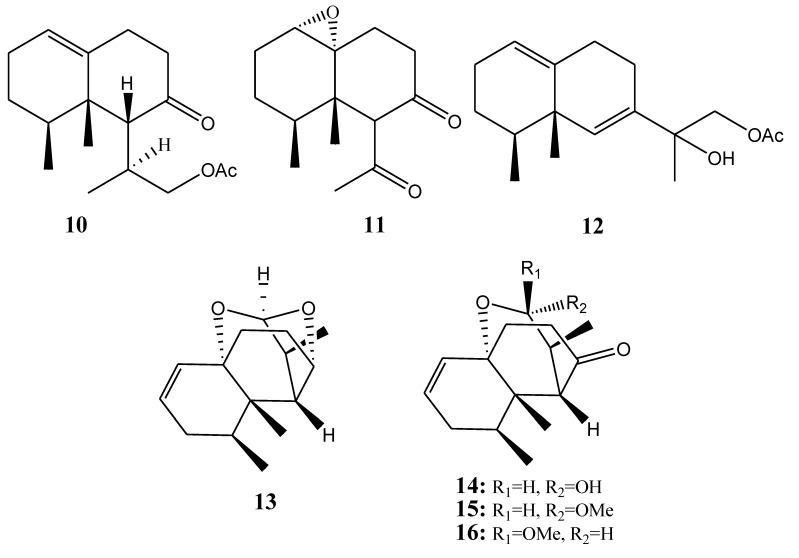
The structures of nardosinane-type sesquiterpenoids (**10**–**16**).

Table 2 summarizes seven nardosinane-type sesquiterpenoids (**10**–**16**) evaluated for *in vitro* anti-inflammatory activity in literature published from 2008 to 2012. The corresponding chemical structures are reported in Figure 2.

**Table 2:** Chemical constituents of nardosinane-type sesquiterpenoids from soft corals of Taiwan.

No.	Name	Sources	Activities *	Reference
**10**	Paralemnolin J	*Paralemnalia thyrsoides*		[15]
**11**	Paralemnolin K	*Paralemnalia thyrsoides*		[15]
**12**	Paralemnolin L	*Paralemnalia thyrsoides*		[15]
**13**	Flavalin A	*Lemnalia flava*	I,C	[16]
**14**	Flavalin B	*Lemnalia flava*		[16]
**15**	Flavalin C	*Lemnalia flava*		[16]
**16**	Flavalin D	*Lemnalia flava*		[16]

* Inhibition of iNOS (I) and COX-2 (C).

#### 2.1.3. Aromadendrane-Type Sesquiterpenoids

**Figure 3:**
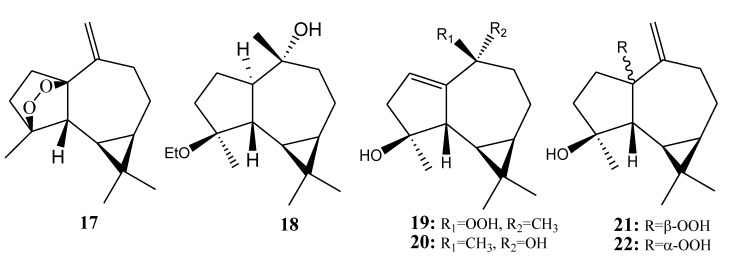
The structures of aromadendrane-type sesquiterpenoids (**17**–**22**).

Table 3 summarizes six aromadendrane-type sesquiterpenoids (**17**–**22**) evaluated for *in vitro* anti-inflammatory activity in literature published from 2008 to 2012. The corresponding chemical structures are reported in Figure 3.

**Table 3:** Chemical constituents of aromadendrane-type sesquiterpenoids from soft corals of Taiwan.

No.	Name	Sources	Activities *	Reference
**17**	Lochmolin A	*Sinularia lochmodes*	C	[17]
**18**	Lochmolin B	*Sinularia lochmodes*	C	[17]
**19**	Lochmolin C	*Sinularia lochmodes*		[17]
**20**	Lochmolin D	*Sinularia lochmodes*		[17]
**21**	Lochmolin E	*Sinularia lochmodes*	C	[17]
**22**	Lochmolin F	*Sinularia lochmodes*	C	[17]

* Inhibition of COX-2 (C).

#### 2.1.4. Selinane- and Oppositane-Type Sesquiterpenoids

**Figure 4:**
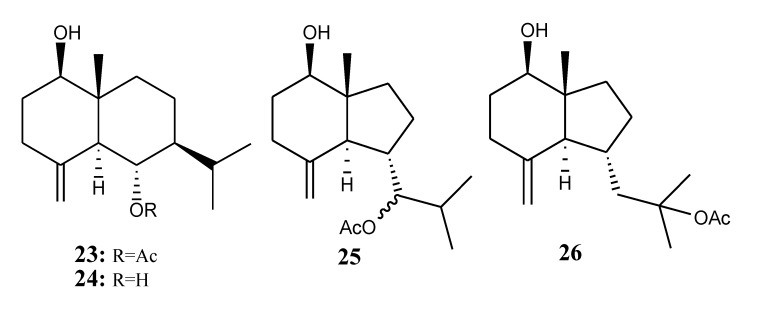
The structures of selinane- and oppositane-type sesquiterpenoids (**23**–**26**).

Table 4 summarizes four selinane- and oppositane-type sesquiterpenoids (**23**–**26**) evaluated for *in vitro* anti-inflammatory activity in literature published from 2008 to 2012. The corresponding chemical structures are reported in Figure 4.

**Table 4:** Chemical constituents of selinane- and oppositane-type sesquiterpenoids from soft corals of Taiwan.

No.	Name	Sources	Activities *	Reference
**23**	1β-Hydroxy-6α-acetoxyeudesm-4(15)-ene	*Sinularia leptoclados*		[18]
**24**	1β,6α-Dihydroxyeudesm-4(15)-ene	*Sinularia leptoclados*	I	[18]
**25**	Leptocladolin A	*Sinularia leptoclados*		[18]
**26**	Leptocladolin B	*Sinularia leptoclados*		[18]

* Inhibition of iNOS (I).

#### 2.1.5. Ylangene-Type Sesquiterpenoids

**Figure 5:**
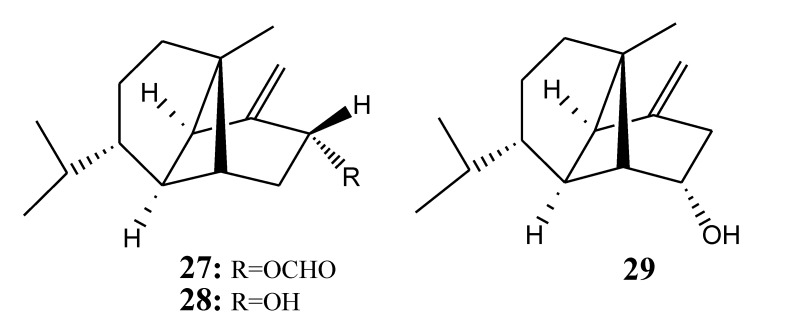
The structures of ylangene-type sesquiterpenoids (**27**–**29**).

Table 5 summarizes three ylangene-type sesquiterpenoids (**27**–**29**) evaluated for *in vitro* anti-inflammatory activity in literature published from 2008 to 2012. The corresponding chemical structures are reported in Figure 5.

**Table 5:** Chemical constituents of ylangene-type sesquiterpenoids from soft corals of Taiwan.

No.	Name	Sources	Activities *	Reference
**27**	(1*S*,2*S*,4*R*,6*S*,7*R*,8*S*)-4α-Formyloxy-β-ylangene	*Lemnalia flava*	I,C	[16]
**28**	Lemnalol	*Lemnalia flava*		[16]
**29**	Isolemnalol	*Lemnalia flava*		[16]

* Inhibition of NOS (I) and COX-2 (C).

#### 2.1.6. Germacrane-Type Sesquiterpenoids

**Figure 6:**
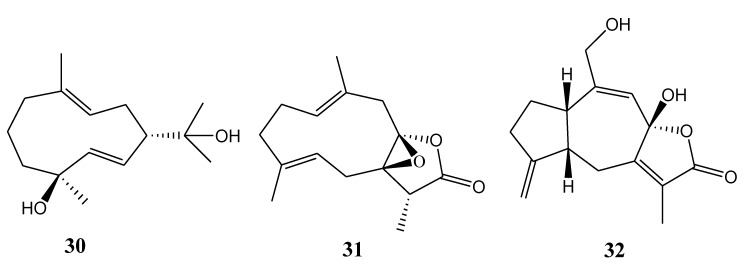
The structures of germacrane-type sesquiterpenoids (**30**–**32**).

Table 6 summarizes three germacrane-type sesquiterpenoids (**30**–**32**) evaluated for *in vitro* anti-inflammatory activity in literature published from 2008 to 2012. The corresponding chemical structures are reported in Figure 6.

**Table 6:** Chemical constituents of germacrane-type sesquiterpenoids from soft corals of Taiwan.

No.	Name	Sources	Activities *	Reference
**30**	Lochmolin G	*Sinularia lochmodes*		[17]
**31**	Menelloide D	*Menella* sp.	E	[19]
**32**	Menelloide E	*Menella* sp.		[20]

* Inhibition of elastase (E).

#### 2.1.7. Other-Type Sesquiterpenoids

**Figure 7:**
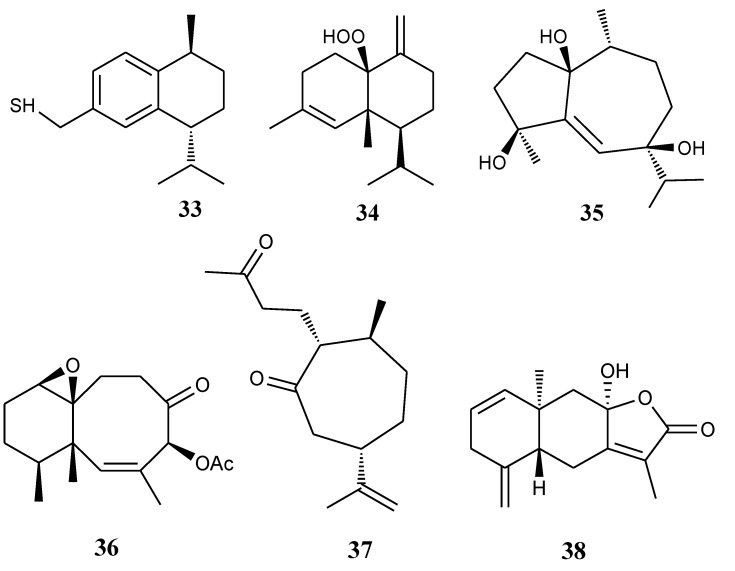
The structures of other-type sesquiterpenoids (**33**–**38**).

Table 7 summarizes six other-type sesquiterpenoids (**33**–**38**) evaluated for *in vitro* anti-inflammatory activity in literature published from 2008 to 2012. The corresponding chemical structures are reported in Figure 7.

**Table 7:** Chemical constituents of other-type sesquiterpenoids from soft corals of Taiwan.

No.	Name	Sources	Activities *	Reference
**33**	Erectathiol	*Nephthea erecta*	I	[21]
**34**	Scabralin A	*Sinularia scabra*	I	[22]
**35**	Leptocladol A	*Sinularia leptoclados*		[23]
**36**	Paralemnolin D	*Paralemnalia thyrsoides*		[15]
**37**	1- *epi*-Chabrolidione A	*Sinularia leptoclados*		[23]
**38**	(–)-Hydroxylindestrenolide	*Menella* sp.	S	[24]

* Inhibition of iNOS (I) and superoxide anion (S).

At a concentration of 10 µM, compounds **1**–**3**, **13**, **24**, **28**, **33** and **34** reduced LPS-induced expression of iNOS in murine macrophage cells [14,15,16,18,21,22]. Compounds **1**, **2**, **13**, **17**, **18**, **21** and **28** suppressed LPS-induced expression of COX-2 in these cells [14,15,16,17]. At 10 µg/mL, compound **38** was shown to slightly inhibit the generation of superoxide anion in FMLP/CB-stimulated human neutrophils, and compound **31** weakly inhibited the release of elastase by activated human neutrophils [19,24]. In addition, an inflammation animal model induced by intraplantar injection of carrageenan into rat hind paws was also used to evaluate *in vivo* anti-inflammatory activity of lemnalol (**28**). Intramuscular injection of **28** (15 mg/kg) significantly inhibited the carrageenan-induced rat paw edema and thermal hyperalgesia behavior. Moreover, lemnalol significantly suppressed the carrageenan-induced expression of iNOS and COX-2 in paw tissue of test rats. Post-intrathecal injection of lemnalol provided an antinociceptive effect in carrageenan-injected rats (1 and 5 μg) [25]. Δ^9(12)^-capnellene-8β,10α-diol (GB9, **1**) and its acetylated derivative, 8α-acetoxy-Δ^9(12)^-capnellene-10α-ol (GB10, **2**) were reported to inhibit the expression of iNOS and COX-2 in BV2 cells post-stimulation by IFN-γ. 

Intraperitoneal administration of GB9 reduced CCI-induced thermal hyperalgesia, suppressed microglial cells activation and COX-2 upregulation in the dorsal horn of the lumbar spinal cord, ipsilateral to the injury. Also, intrathecal administration of GB9 and GB10 suppressed activities of CCl-induced nociceptive sensitization and thermal hyperalgesia [26]. The above findings suggest that some of these compounds may warrant systematic investigation for future development as immune-modifiers. 

### 2.2. Diterpenoids

#### 2.2.1. Cembrane-Based Diterpenoids

**Figure 8:**
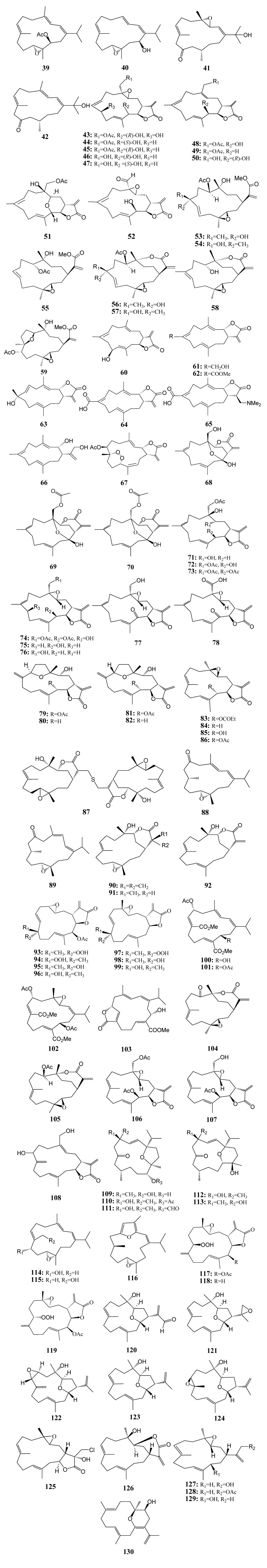
The structures of cembrane-based diterpenoids (**39**–**130**).

Table 8 summarizes 92 cembrane-based diterpenoids (**39**–**130**) evaluated for *in vitro* anti-inflammatory activity in literature published from 2008 to 2012. The corresponding chemical structures are reported in Figure 8.

**Table 8:** Chemical constituents of cembrane-based diterpenoids from soft corals of Taiwan.

	Name	Sources	Activities *	Reference
**39**	Gibberosene B	*Sinularia gibberosa*	I,C	[27]
**40**	(+)-11,12-Epoxysarcophytol A	*Sinularia gibberosa*		[27]
**41**	Grandilobatin B	*Sinularia grandilobata*		[28]
**42**	Grandilobatin D	*Sinularia grandilobata*	I	[28]
**43**	Durumolide A	*Lobophytum durum*	I,C	[29]
**44**	13*S*-Hydroxylobolide	*Lobophytum durum*	I,C	[29]
**45**	13*R*-Hydroxylobolide	*Lobophytum durum*	I	[29]
**46**	Deacetyl-13-hydroxylobolide	Lobophytum durum	I,C	[27]
**47**	(7*E*,11*E*)-13,18-Dihydroxy-3,4-epoxy-7,11,15(17)-cembratrien-16,14-olide	Lobophytum durum	I,C	[27]
**48**	Durumolide B	*Lobophytum durum*	I	[28]
**49**	(3*E*,7*E*,11*E*)-18-Acetoxy-3,7,11,15(17)-cembratetraen-16,14-olide	*Lobophytum durum*	I,C	[28]
**50**	Durumolide C	*Lobophytum durum*	I,C	[29]
**51**	Durumolide D	*Lobophytum durum*	I	[29]
**52**	Durumolide E	*Lobophytum durum*	I	[29]
**53**	Granosolide C	*Sinularia granosa*		[30]
**54**	Querciformolide E	*Sinularia querciformis*	I	[30]
**55**	Granosolide D	*Sinularia granosa*	I	[30]
**56**	Flexibilisolide A	*Sinularia granosa*	I	[30]
**57**	Flexilarin	*Sinularia granosa*	I	[30]
**58**	Sinulariolide	*Sinularia granosa*	I	[30]
**59**	Sinulaflexiolide E	*Sinularia granosa*		[30]
**60**	Crassumolide A	*Lobophytum crassum*	I,C	[31]
**61**	Crassumolide B	*Lobophytum crassum*	I	[31]
**62**	Crassumolide C	*Lobophytum crassum*	I,C	[31]
**63**	Crassumolide F	*Lobophytum crassum*	I	[31]
**64**	Lobohedleolide	*Lobophytum crassum*	I,C	[31]
**65**	17-Dimethylaminolobohedleolide	*Lobophytum crassum*	I	[31]
**66**	Sinulariol A	*Lobophytum crassum*	I,C	[31]
**67**	Dentivulatolide	*Lobophytum crassum*	I,C	[31]
**68**	Durumhemiketalolide A	*Lobophytum durum*	I,C	[32]
**69**	Durumhemiketalolide B	*Lobophytum durum*	I	[32]
**70**	Durumhemiketalolide C	*Lobophytum durum*	I,C	[32]
**71**	Durumolide F	*Lobophytum durum*	I,C	[33]
**72**	Durumolide G	*Lobophytum durum*	I	[33]
**73**	Durumolide H	*Lobophytum durum*	I	[33]
**74**	Durumolide I	*Lobophytum durum*	I	[33]
**75**	Durumolide J	*Lobophytum durum*	I	[33]
**76**	Sinularolide D	*Lobophytum durum*	I	[33]
**77**	Durumolide K	*Lobophytum durum*	I,C	[33]
**78**	Durumolide L	*Lobophytum durum*	I	[33]
**79**	Sarcocrassocolide A	*Sarcophyton crassocaule*	I	[34]
**80**	Sarcocrassocolide C	*Sarcophyton crassocaule*	I	[34]
**81**	Sarcocrassocolide B	*Sarcophyton crassocaule*	I	[34]
**82**	Sarcocrassocolide D	*Sarcophyton crassocaule*	I	[34]
**83**	Sarcocrassocolide E	*Sarcophyton crassocaule*	I	[34]
**84**	Sarcocrassolide	*Sarcophyton crassocaule*	I,C	[34]
**85**	Sinularolide	*Sarcophyton crassocaule*	I	[34]
**86**	13-Acetoxysarcocrassolide	*Sarcophyton crassocaule*	I	[34]
**87**	Thioflexibilolide A	*Sinularia flexibilis*	I,C	[35]
**88**	Triangulene A	*Sinularia triangular*		[36]
**89**	Triangulene B	*Sinularia triangular*		[36]
**90**	Sinularin	*Sinularia triangular*	I	[36]
**91**	Dihydrosinularin	*Sinularia triangular*	I,C	[36]
**92**	(−)14-Deoxycrassin	*Sinularia triangular*	I,C	[36]
**93**	Sarcocrassocolide F	*Sarcophyton crassocaule*	I	[37]
**94**	Sarcocrassocolide G	*Sarcophyton crassocaule*	I	[37]
**95**	Sarcocrassocolide H	*Sarcophyton crassocaule*	I	[37]
**96**	Sarcocrassocolide I	*Sarcophyton crassocaule*	I,C	[37]
**97**	Sarcocrassocolide J	*Sarcophyton crassocaule*	I	[37]
**98**	Sarcocrassocolide K	*Sarcophyton crassocaule*	I	[37]
**99**	Sarcocrassocolide L	*Sarcophyton crassocaule*	I	[37]
**100**	Sarcophytolin A	*Lobophytum sarcophytoides*	I	[38]
**101**	Sarcophytolin B	*Lobophytum sarcophytoides*	I	[38]
**102**	Sarcophytolin C	*Lobophytum sarcophytoides*		[38]
**103**	Sarcophytolin D	*Lobophytum sarcophytoides*	I	[38]
**104**	11-Dehydrosinulariolide	*Sinularia discrepans*	I,C	[39]
**105**	11-*epi*-Sinulariolide acetate	*Sinularia discrepans*	I,C	[39]
**106**	Crassumolide G	*Lobophytum crassum*	I	[40]
**107**	Crassumolide H	*Lobophytum crassum*	I	[40]
**108**	Crassumolide I	*Lobophytum crassum*	I	[40]
**109**	Crassarine A	*Sinularia crassa*		[41]
**110**	Crassarine B	*Sinularia crassa*		[41]
**111**	Crassarine C	*Sinularia crassa*		[41]
**112**	Crassarine D	*Sinularia crassa*		[41]
**113**	Crassarine E	*Sinularia crassa*		[41]
**114**	Crassarine F	*Sinularia crassa*	C	[41]
**115**	Crassarine G	*Sinularia crassa*		[41]
**116**	Crassarine H	*Sinularia crassa*	I	[41]
**117**	Sarcocrassocolide M	*Sarcophyton crassocaule*	I	[42]
**118**	Sarcocrassocolide N	*Sarcophyton crassocaule*	I	[42]
**119**	Sarcocrassocolide O	*Sarcophyton crassocaule*	I	[42]
**120**	Culobophylin A	*Lobophytum crassum*		[43]
**121**	Culobophylin B	*Lobophytum crassum*		[43]
**122**	Culobophylin C	*Lobophytum crassum*		[43]
**123**	Lobophylin B	*Lobophytum crassum*		[43]
**124**	Lobophylin A	*Lobophytum crassum*		[43]
**125**	Lobocrassin A	*Lobophytum crassum*		[44]
**126**	Lobocrassin B	*Lobophytum crassum*	S,E	[44]
**127**	Lobocrassin C	*Lobophytum crassum*		[44]
**128**	Lobocrassin D	*Lobophytum crassum*		[44]
**129**	Lobocrassin E	*Lobophytum crassum*		[44]
**130**	Lobocrassin F	*Lobophytum crassum*	E	[20]

* Inhibition of iNOS (I), COX-2 (C), superoxide anion (S) and elastase (E).

At the concentration of 10 µM, compounds **39**, **42**–**52**, **54**–**58**, **60**–**87**, **90**–**101**, **103**–**108** and **116**–**119** reduced LPS-induced expression of iNOS in murine macrophage (RAW264.7) cells [27,28,29,30,31,32,33,34,35,36,37,38,39,40,41,42]. Compounds **39**, **43**, **44**, **46**, **47**, **49**, **50**, **62**, **64**, **66**–**68**, **70**, **71**, **77**, **84**, **87**, **91**, **92**, **96**, **104**, **105** and **114** suppressed LPS-induced expression of COX-2 in these cells [27,29,31,32,33,34,35,36,37,39,41]. At 10 µg/mL, compound **126** inhibited the generation of superoxide anion and the release of elastase in human neutrophils [44]. Compound **130** inhibited the release of elastase by activated human neutrophils [24]. For *in vivo* anti-inflammatory activities, subcutaneous (s.c.) administration of sinularin (**90**) (80 mg/kg) significantly inhibited carrageenan-induced nociceptive behaviors as well as carrageenan-induced activation of microglial and astrocyte, and the iNOS expression in the dorsal horn of the lumbar spinal cord [45]. Due to its promising anti-inflammatory profile, sinularin may warrant future exploration as a lead compound for immune-/inflammation-modulation.

#### 2.2.2. Eunicellin-Based Diterpenoids

**Figure 9:**
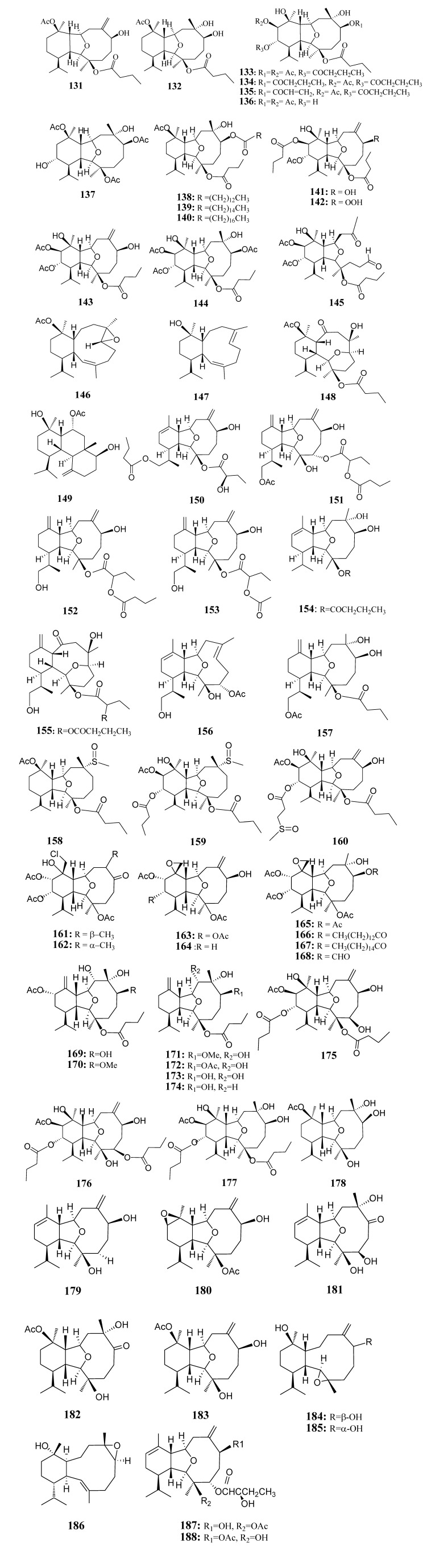
The structures of cembrane-based diterpenoids (**131**–**188**).

Table 9 summarizes 58 eunicellin-based diterpenoids (**131**–**188**) evaluated for *in vitro* anti-inflammatory activity in literature published from 2008 to 2012. The corresponding chemical structures are reported in Figure 9.

**Table 9:** Chemical constituents of eunicellin-based diterpenoids from soft corals of Taiwan.

No.	Name	Sources	Activities *	Reference
**131**	Simplexin A	*Klyxum simplex*	I	[46]
**132**	Simplexin B	*Klyxum simplex*		[46]
**133**	Simplexin C	*Klyxum simplex*		[46]
**134**	Simplexin D	*Klyxum simplex*	I	[46]
**135**	Simplexin E	*Klyxum simplex*	I,C	[46]
**136**	Simplexin F	*Klyxum simplex*		[46]
**137**	Simplexin I	*Klyxum simplex*		[46]
**138**	Klysimplexin I	*Klyxum simplex*		[47]
**139**	Klysimplexin J	*Klyxum simplex*	I	[47]
**140**	Klysimplexin K	*Klyxum simplex*	I	[47]
**141**	Klysimplexin L	*Klyxum simplex*	I	[47]
**142**	Klysimplexin M	*Klyxum simplex*	I	[47]
**143**	Klysimplexin N	*Klyxum simplex*	I	[47]
**144**	Klysimplexin O	*Klyxum simplex*		[47]
**145**	Klysimplexin P	*Klyxum simplex*		[47]
**146**	Klysimplexin Q	*Klyxum simplex*		[47]
**147**	Klysimplexin R	*Klyxum simplex*	I	[47]
**148**	Klysimplexin S	*Klyxum simplex*	I,C	[47]
**149**	Klysimplexin T	*Klyxum simplex*		[47]
**150**	Hirsutalin A	*Cladiella hirsuta*		[48]
**151**	Hirsutalin B	*Cladiella hirsuta*	I,C	[48]
**152**	Hirsutalin C	*Cladiella hirsuta*	I	[48]
**153**	Hirsutalin D	*Cladiella hirsuta*	I	[48]
**154**	Hirsutalin E	*Cladiella hirsuta*		[48]
**155**	Hirsutalin F	*Cladiella hirsuta*		[48]
**156**	Hirsutalin G	*Cladiella hirsuta*		[48]
**157**	Hirsutalin H	*Cladiella hirsuta*	I	[48]
**158**	Klysimplexin sulfoxide A	*Klyxum simplex*	I	[49]
**159**	Klysimplexin sulfoxide B	*Klyxum simplex*	I	[49]
**160**	Klysimplexin sulfoxide C	*Klyxum simplex*	I,C	[49]
**161**	Lymollin A	*Klyxum molle*		[50]
**162**	Lymollin B	*Klyxum molle*	I	[50]
**163**	Lymollin C	*Klyxum molle*	I,C	[50]
**164**	Lymollin D	*Klyxum molle*	I,C	[50]
**165**	Lymollin E	*Klyxum molle*	I	[50]
**166**	Lymollin F	*Klyxum molle*	I,C	[50]
**167**	Lymollin G	*Klyxum molle*	I,C	[50]
**168**	Lymollin H	*Klyxum molle*	I,C	[50]
**169**	Krempfielin A	*Cladiella krempfi*		[51]
**170**	Krempfielin D	*Cladiella krempfi*	I	[51]
**171**	Krempfielin B	*Cladiella krempfi*	I	[51]
**172**	krempfielin C	*Cladiella krempfi*	I	[51]
**173**	Litophynol B	*Cladiella krempfi*	I	[51]
**174**	(1R*,2R*,3R*,6S*,7S*,9R*,10R*,14R*)3-Butanoyloxycladiell-11(17)-en-6,7-diol	*Cladiella krempfi*	I	[51]
**175**	Klysimplexin U	*Klyxum simplex*		[52]
**176**	Klysimplexin V	*Klyxum simplex*		[52]
**177**	Klysimplexin W	*Klyxum simplex*		[52]
**178**	Klysimplexin X	*Klyxum simplex*		[52]
**179**	Cladieunicellin A	*Cladiella* sp.	S,E	[53]
**180**	Cladieunicellin C	*Cladiella* sp.		[53]
**181**	Cladieunicellin D	*Cladiella* sp.		[53]
**182**	Cladieunicellin E	*Cladiella* sp.		[53]
**183**	Cladieunicellin G	*Cladiella* sp.	S,E	[54]
**184**	6-*epi*-Cladieunicellin F	*Cladiella* sp.		[54]
**185**	Cladieunicellin F	*Cladiella* sp.	S,E	[54]
**186**	(–)-Solenopodin C	*Cladiella* sp.		[55]
**187**	Cladielloide A	*Cladiella* sp.		[56]
**188**	Cladielloide B	*Cladiella* sp.	S,E	[56]

* Inhibition of iNOS (I), COX-2 (C), superoxide anion (S) and elastase (E).

#### 2.2.3. Briarane-based Diterpenoids

**Figure 10:**
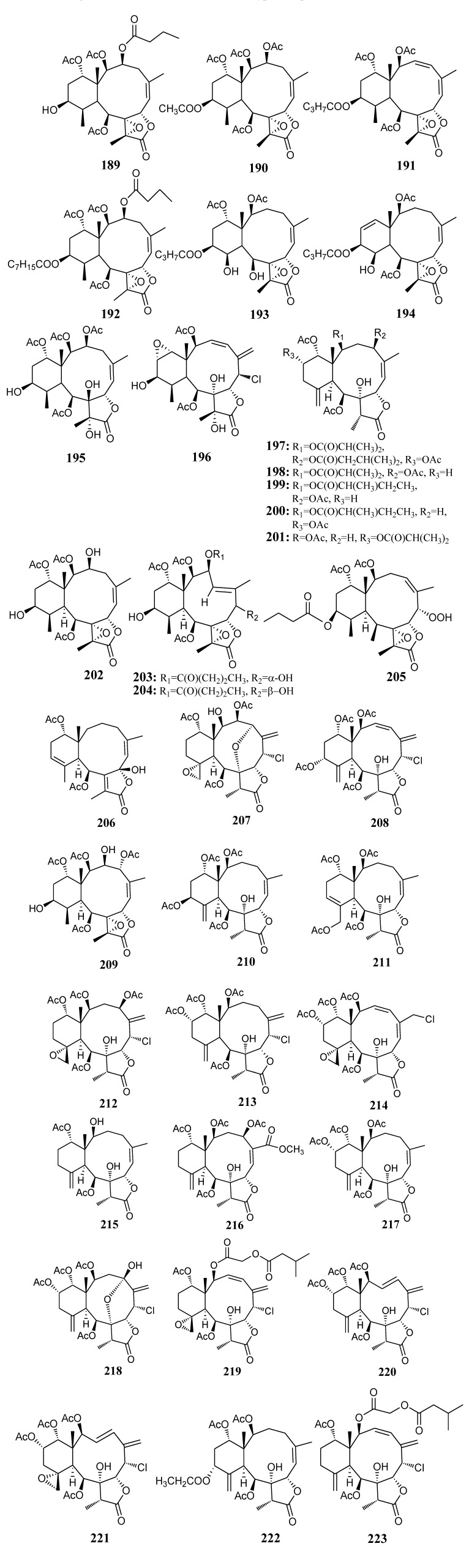
The structures of briarane-type diterpenoids (**189**–**223**).

Table 10 summarizes 35 briarane-based diterpenoids (**189**–**223**) evaluated for *in vitro* anti-inflammatory activity in literature published from 2008 to 2012. The corresponding chemical structures are reported in Figure 10.

**Table 10:** Chemical constituents of briarane-type diterpenoids from soft corals of Taiwan.

No.	Name	Sources	Activities *	Reference
**189**	Excavatolide B	*Briareum excavatum*		[57]
**190**	Excavatolide K	*Briareum excavatum*		[57]
**191**	Excavatolide F	*Briareum excavatum*		[57]
**192**	Briaexcavatolide R	*Briareum excavatum*		[57]
**193**	Excavatolide Z	*Briareum excavatum*		[57]
**194**	Briaexcavatolide B	*Briareum excavatum*		[57]
**195**	Briaexcavatolide K	*Briareum excavatum*		[57]
**196**	Briaexcavatolide H	*Briareum excavatum*		[57]
**197**	Junceol D	*Junceella juncea*		[58]
**198**	Junceol E	*Junceella juncea*	S	[58]
**199**	Junceol F	*Junceella juncea*	S	[58]
**200**	Junceol G	*Junceella juncea*	S	[58]
**201**	Junceol H	*Junceella juncea*	S	[58]
**202**	Excavatoid L	*Briareum excavatum*	S,E	[59]
**203**	Excavatoid M	*Briareum excavatum*	S,E	[59]
**204**	Excavatoid N	*Briareum excavatum*	S,E	[59]
**205**	Briarenolide F	*Briareum* sp.	S	[60]
**206**	Briarenolide G	*Briareum* sp.		[60]
**207**	Fragilide J	*Ellisella robusta*	E	[61]
**208**	Robustolide L	*Ellisella robusta*	S	[61]
**209**	Briaexcavatin P	*Briareum excavatum*	S	[62]
**210**	Frajunolide L	*Junceella fragilis*	S,E	[63]
**211**	Frajunolide M	*Junceella fragilis*		[63]
**212**	Frajunolide N	*Junceella fragilis*	E	[63]
**213**	Frajunolide O	*Junceella fragilis*	S,E	[63]
**214**	Juncenolide M	*Junceella juncea*		[64]
**215**	Juncenolide N	*Junceella juncea*	E	[64]
**216**	Juncenolide O	*Junceella juncea*	S,E	[64]
**217**	Frajunolide E	*Junceella fragilis*	S,E	[65]
**218**	Frajunolide F	*Junceella fragilis*		[65]
**219**	Frajunolide G	*Junceella fragilis*		[65]
**220**	Frajunolide H	*Junceella fragilis*		[65]
**221**	Frajunolide I	*Junceella fragilis*		[65]
**222**	Frajunolide J	*Junceella fragilis*	S,E	[65]
**223**	Frajunolide K	*Junceella fragilis*		[65]

* Inhibition of superoxide anion (S) and elastase (E).

#### 2.2.4. Verticillane-Based Diterpenoids

**Figure 11:**
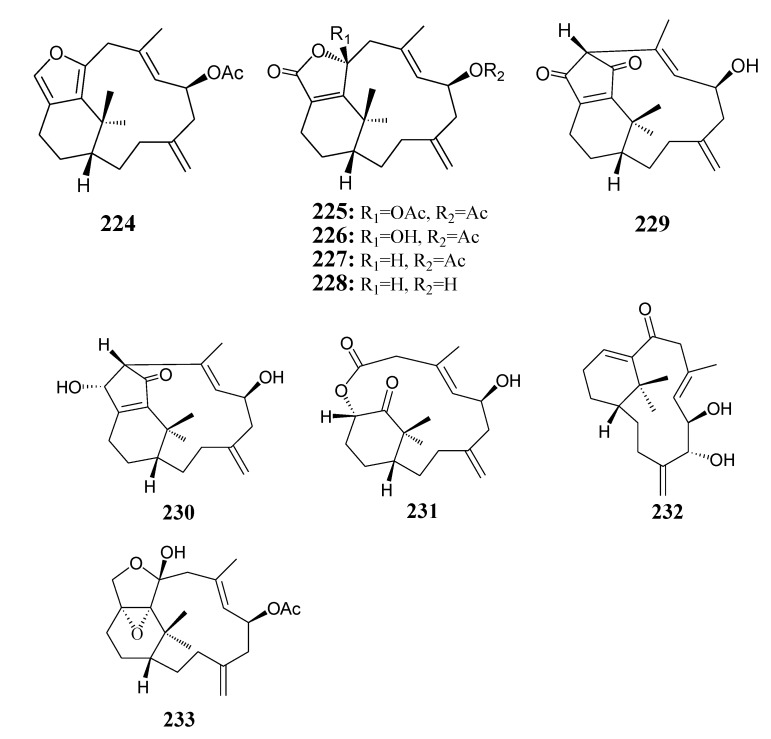
The structures of verticillane-based diterpenoids (**224**–**233**).

Table 11 summarizes 10 verticillane-based diterpenoids (**224**–**233**) evaluated for *in vitro* anti-inflammatory activity in literature published from 2008 to 2012. The corresponding chemical structures are reported in Figure 11.

**Table 11:** Chemical constituents of verticillane-type diterpenoids from soft corals of Taiwan.

No.	Name	Sources	Activities *	Reference
**224**	Cespitularin R	*Cespitularia hypotentaculata*		[66]
**225**	Cespitularin S	*Cespitularia hypotentaculata*	I,C	[66]
**226**	Cespitularin J	*Cespitularia hypotentaculata*		[66]
**227**	Cesputularin K	*Cespitularia hypotentaculata*	I	[66]
**228**	Cespitularin M	*Cespitularia hypotentaculata*		[66]
**229**	Cespitularin I	*Cespitularia hypotentaculata*	I	[66]
**230**	Cespitularin F	*Cespitularia hypotentaculata*	I	[66]
**231**	Cespitularin Q	*Cespitularia hypotentaculata*		[66]
**232**	Cespitulin E	*Cespitularia taenuate*	S,E	[67]
**233**	Cespitulin G	*Cespitularia taenuate*	S,E	[67]

* Inhibition of iNOS (I), COX-2 (C), superoxide anion (S) and elastase (E).

#### 2.2.5. Norditerpenoids

**Figure 12:**
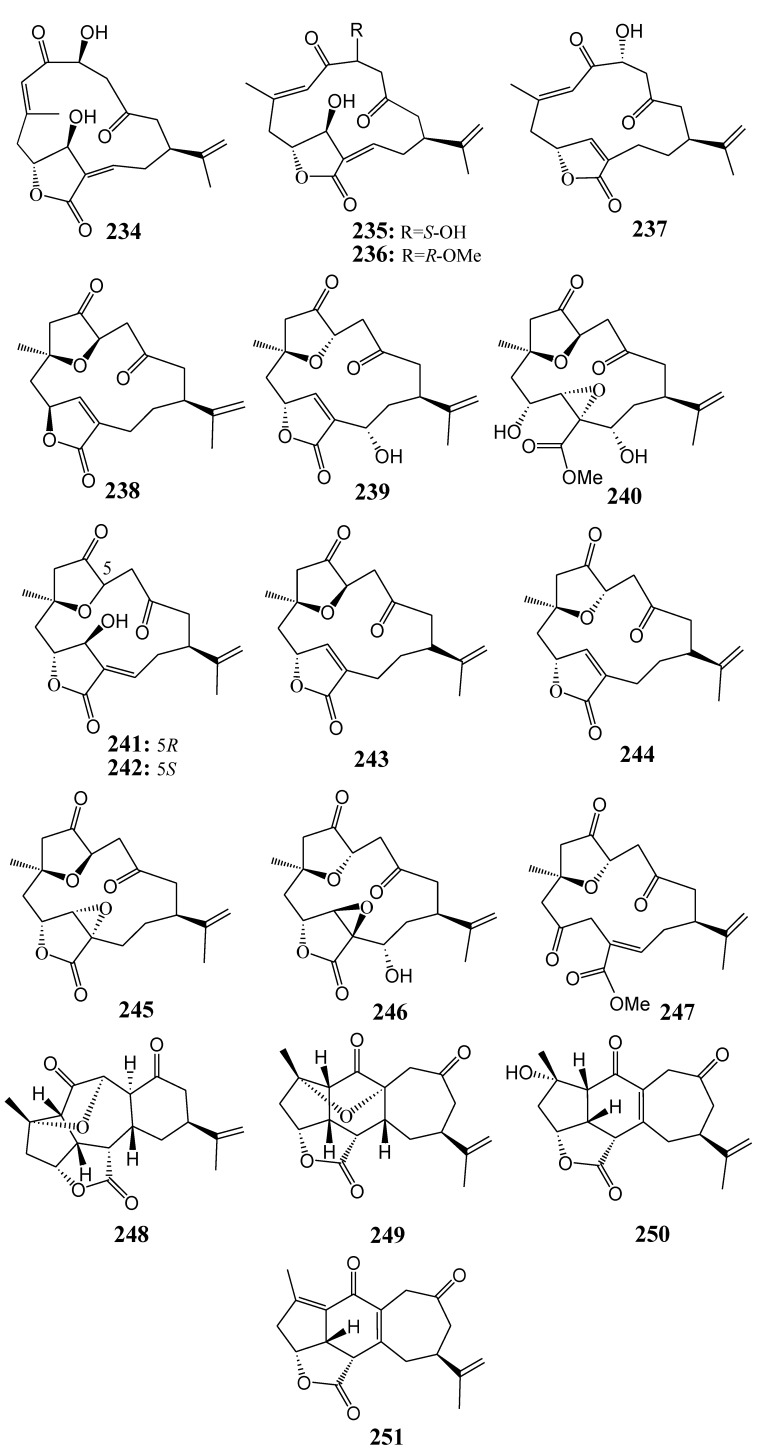
The structures of norditerpenoids (**234**–**251**).

Table 12 summarizes 18 norditerpenoids (**234**–**251**) evaluated for *in vitro* anti-inflammatory activity in literature published from 2008 to 2012. The corresponding chemical structures are reported in Figure 12.

**Table 12:** Chemical constituents of norditerpenoids from soft corals of Taiwan.

No.	Name	Sources	Activities *	Reference
**234**	Gyrosanolide A	*Sinularia gyrosa*		[68]
**235**	Gyrosanolide B	*Sinularia gyrosa*	I	[68]
**236**	Gyrosanolide C	*Sinularia gyrosa*	I	[68]
**237**	Gyrosanolide D	*Sinularia gyrosa*		[68]
**238**	Gyrosanolide E	*Sinularia gyrosa*		[68]
**239**	Gyrosanolide F	*Sinularia gyrosa*	I	[68]
**240**	Gyrosanin A	*Sinularia gyrosa*	I	[68]
**241**	(1 S*,5R*,8S*,10R*,11S*)-11-Hydroxyl-1-isopropenyl-8-methyl-3,6-dioxo-5,8-epoxycyclotetradec-12-ene-10,12-carbonlactone	*Sinularia gyrosa*	I	[68]
**242**	(1 S*,5S*,8S*,10R*,11S*)-11-Hydroxyl-1-isopropenyl-8-methyl-3,6-dioxo-5,8-epoxycyclotetradec-12-ene-10,12-carbonlactone	*Sinularia gyrosa*	I	[68]
**243**	Norcembrene	*Sinularia gyrosa*		[68]
**244**	*epi*-Norcembrene	*Sinularia gyrosa*		[68]
**245**	Leptocladolide B	*Sinularia gyrosa*	I	[68]
**246**	Scabrolide D	*Sinularia gyrosa*	I	[68]
**247**	Norcembrene	*Sinularia gyrosa*		[68]
**248**	Ineleganolide	*Sinularia gyrosa*		[68]
**249**	Sinulochemodin C	*Sinularia gyrosa*		[68]
**250**	Scabrolide A	*Sinularia gyrosa*		[68]
**251**	Yanarolide	*Sinularia gyrosa*		[68]

* Inhibition of iNOS (I).

#### 2.2.6. Xenicane-Type Diterpenoids

**Figure 13:**
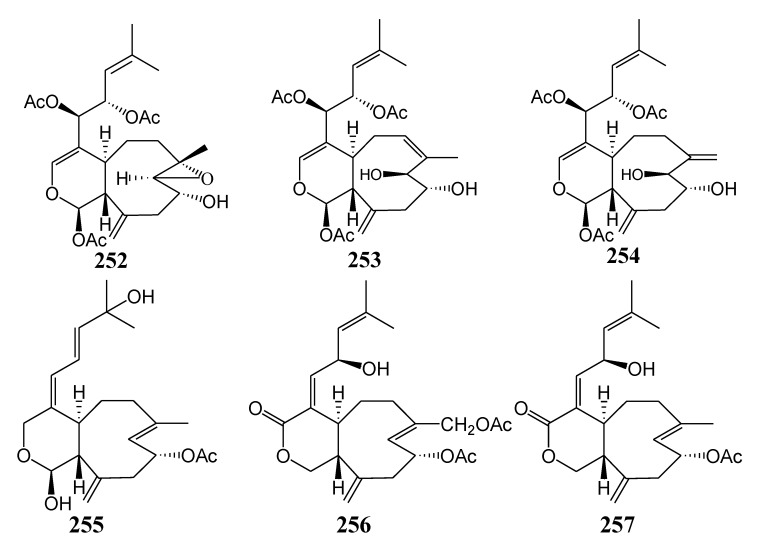
The structures of xenicane-type diterpenoids (**252**–**257**).

Table 13 summarizes six xenicane-type diterpenoids (**252**–**257**) evaluated for *in vitro* anti-inflammatory activity in literature published from 2008 to 2012. The corresponding chemical structures are reported in Figure 13.

**Table 13:** Chemical constituents of xenicane-type diterpenoids from soft corals of Taiwan.

No.	Name	Sources	Activities *	Reference
**252**	Asterolaurin A	*Asterospicularia laurae*		[69]
**253**	Asterolaurin B	*Asterospicularia laurae*		[69]
**254**	Asterolaurin C	*Asterospicularia laurae*		[69]
**255**	Asterolaurin D	*Asterospicularia laurae*	S,E	[69]
**256**	Asterolaurin E	*Asterospicularia laurae*		[69]
**257**	Asterolaurin F	*Asterospicularia laurae*		[69]

* Inhibition of superoxide anion (S) and elastase (E).

#### 2.2.7. Other-Type Diterpenoids

**Figure 14:**
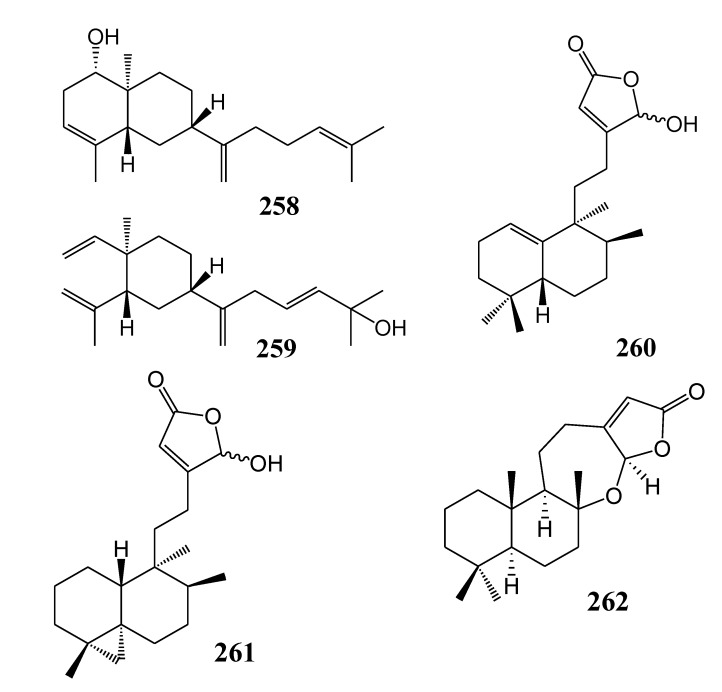
The structures of other type diterpenoids (**258**–**262**).

Table 14 summarizes five other-type diterpenoids (**258**–**262**) evaluated for *in vitro* anti-inflammatory activity in literature published from 2008 to 2012. The corresponding chemical structures are reported in Figure 14.

**Table 14:** Chemical constituents of other type diterpenoids from soft corals of Taiwan.

No.	Name	Sources	Activities *	Reference
**258**	Gyrosanol A	*Sinularia gyrosa*	C	[70]
**259**	Gyrosanol B	*Sinularia gyrosa*	C	[70]
**260**	Echinohalimane A	*Echinomuricea* sp.	E	[71]
**261**	Echinoclerodane A	*Echinomuricea* sp.	S,E	[72]
**262**	Echinolabdane A	*Echinomuricea* sp.		[73]

* Inhibition of COX-2 (C), superoxide anion (S) and elastase (E).

At a concentration of 10 μM, compounds **131**, **133**, **134**, **139**, **140**–**143**, **147**, **148**, **151**–**153**, **157**–**160**, **162**–**168**, **170** ceramide and cerebrosides **174**, **225**, **229**, **230**, **235**, **236**, **239**–**242**, **244**, **245**, **258** and **259** reduced LPS-induced expression of iNOS in murine macrophage cells [46,47,48,49,50,51,66,68,70]. Compounds **134**, **148**, **151**, **160**, **163**, **164**, **166**–**168**, **225**, **258** and **259** suppressed the LPS-induced expression of COX-2 in these cells [46,47,48,49,50,66,70]. At 10 µg/mL, compounds **180**, **184**, **186**, **188**, **198**–**205**, **208**–**210**, **213**, **216**, **217**, **222**, **232**, **233**, **255** and **261** inhibited the generation of superoxide anion by activated human neutrophils [54,55,56,58,59,60,61,62,63,64,65,67,69,70,72]. Compounds **180**, **184**, **186**, **188**, **202**–**204**, **207**, **210**, **212**, **213**, **215**–**217**, **222**, **232**, **233**, **255**, **260** and **261** inhibited the release of elastase from these activated human neutrophils [53,54,55,56,59,61,63,65,67,69,71,72]. These results provided useful baseline information on the immune-regulatory and anti-oxidant activities of various marine diterpenoids. Compound **184**, as **185** epimer at C-6, was showed to be more potent in the inhibition of the generation of superoxide anion and in inducing the release of elastase by active human neutrophils, suggesting that the stereochemistry at C-6 may play a key role in the above biological effects [54].

The briarane-type diterpenoid excavatolide B (**189**) has been demonstrated to significantly inhibit TPA-induced cutaneous inflammation activities in mice, including those related to vascular permeability, edema, and TPA-induced expression of iNOS, COX-2 and matrixmetalloproteinase-9. Excavatolide B also suppressed LPS-induced expression of TNF-α and IL-6 in mouse bone marrow derived dendritic cells (BMDCs) [57]. Also, excavatolide F (**191**), K (**190**) and Z (**193**) and briaexcavatolide B (**194**), H (**196**), K (**195**) and R (**192**) exhibited a broad spectrum of activity in inhibition of LPS-induced expression of IL-6 in BMDCs [57]. A study on the structure-activity relationship between the structures of the briarane-type diterpenoids and their inhibition of IL-6 expression in BMDCs revealed that the eight 17-epoxide of briarane-type diterpenoids may play an important role in the inhibition of IL-6 expression in specific immune cells [57]. Replacement of the C-12 hydroxyl group with long esters in briarane-type diterpenoids decreased the inhibition of IL-6 expression [57].

### 2.3. Steroids

**Figure 15:**
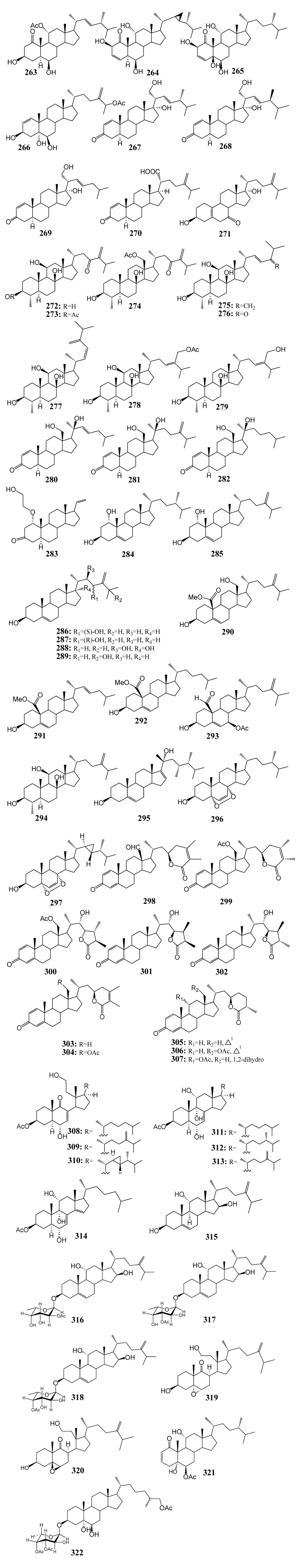
The structures of steroids (**263**–**322**).

Table 15 summarizes 60 steroids (**263**–**322**) evaluated for *in vitro* anti-inflammatory activity in literature published from 2008 to 2012. The corresponding chemical structures are reported in Figure 15.

**Table 15:** Chemical constituents of steroids from soft corals of Taiwan.

No.	Name	Sources	Activities *	Reference
**263**	Stoloniferone R	*Clavularia viridis*		[74]
**264**	Stoloniferone S	*Clavularia viridis*	I	[74]
**265**	Stoloniferone T	*Clavularia viridis*	I,C	[74]
**266**	(25*S*)-24-Methylenecholestane-3β,5α,6β-triol-26-acetate	*Clavularia viridis*	I,C	[74]
**267**	Griffinisterone A	*Nephthea griffini*	I	[75]
**268**	Griffinisterone B	*Nephthea griffini*	I	[75]
**269**	Griffinisterone C	*Nephthea griffini*	I	[75]
**270**	Griffinisterone D	*Nephthea griffini*	I	[75]
**271**	Chabrosterol	*Nephthea chabroli*	I,C	[21]
**272**	Nebrosteroid A	*Nephthea chabroli*	I	[76]
**273**	Nebrosteroid B	*Nephthea chabroli*	I	[76]
**274**	Nebrosteroid C	*Nephthea chabroli*	I	[76]
**275**	Nebrosteroid D	*Nephthea chabroli*	I,C	[76]
**276**	Nebrosteroid F	*Nepthea chabroli*	I,C	[76]
**277**	Nebrosteroid E	*Nepthea chabroli*		[76]
**278**	Nebrosteroid G	*Nepthea chabroli*	I,C	[76]
**279**	Nebrosteroid H	*Nepthea chabroli*	I	[76]
**280**	Griffinisterone F	*Dendronephthya griffini*	I,C	[77]
**281**	Griffinisterone G	*Dendronephthya griffini*	I,C	[77]
**282**	Griffinisterone H	*Dendronephthya griffini*	I	[77]
**283**	Griffinipregnone	*Dendronephthya griffini*	I,C	[77]
**284**	1α,3β-Dihydroxy-24*S*-methylcholesta-5-ene	*Sinularia sp.*	I,C	[78]
**285**	1α,3β-Dihydroxy-24-methylenecholesta-5-ene	*Sinularia sp.*	I,C	[78]
**286**	5,24(28)-Ergostadien-3β,23*S*-diol	*Nephthea erecta*	I,C	[79]
**287**	5,24(28)-Ergostadien-3β,23*R*-diol	*Nephthea erecta*	I	[79]
**288**	(22*S*)-5,24(28)-Ergostadien-3β,17α,22-triol	*Nephthea erecta*	I,C	[79]
**289**	Ergostanoid	*Nephthea erecta*	I	[79]
**290**	Nebrosteroid I	*Nephthea chabroli*	I,C	[80]
**291**	Nebrosteroid J	*Nephthea chabroli*	I,C	[80]
**292**	Nebrosteroid K	*Nephthea chabroli*		[80]
**293**	Nebrosteroid L	*Nephthea chabroli*	I,C	[80]
**294**	Nebrosteroid M	*Nephthea chabroli*	IC	[80]
**295**	Sarcophytosterol	*Lobophytum sarcophytoides*		[38]
**296**	5α,8α-Epidioxy-24-methylcholesta-6-en-3β-ol	*Lobophytum sarcophytoides*		[38]
**297**	5α,8α-Epidioxy-22,23-methylene-24-methylcholest-6-en-3β-ol	*Lobophytum sarcophytoides*	I	[38]
**298**	Paraminabeolide A	*Paraminabea acronocephala*	I	[81]
**299**	Paraminabeolide B	*Paraminabea acronocephala*	I	[81]
**300**	Paraminabeolide C	*Paraminabea acronocephala*	I	[81]
**301**	Paraminabeolide D	*Paraminabea acronocephala*	I	[81]
**302**	Paraminabeolide E	*Paraminabea acronocephala*		[81]
**303**	Minabeolide-1	*Paraminabea acronocephala*	I,C	[81]
**304**	Minabeolide-2	*Paraminabea acronocephala*	I,C	[81]
**305**	Minabeolide-4	*Paraminabea acronocephala*	I,C	[81]
**306**	Minabeolide-5	*Paraminabea acronocephala*	I,C	[81]
**307**	Minabeolide-8	*Paraminabea acronocephala*		[81]
**308**	Hirsutosterol A	*Cladiella hirsuta*		[82]
**309**	Hirsutosterol B	*Cladiella hirsuta*		[82]
**310**	Hirsutosterol C	*Cladiella hirsuta*		[82]
**311**	Hirsutosterol D	*Cladiella hirsuta*		[82]
**312**	Hirsutosterol E	*Cladiella hirsuta*		[82]
**313**	Hirsutosterol F	*Cladiella hirsuta*		[82]
**314**	Hirsutosterol G	*Cladiella hirsuta*		[82]
**315**	Crassarosterol A	*Sinularia crassa*		[83]
**316**	Crassarosteroside A	*Sinularia crassa*	I	[83]
**317**	Crassarosteroside B	*Sinularia crassa*	I	[83]
**318**	Crassarosteroside C	*Sinularia crassa*	I	[83]
**319**	8αH-3β*,*11-Dihydroxy-5α,6α-expoxy-24-methylene-9,11-secocholestan-9-one	*Sinularia granosa*	I,C	[84]
**320**	3β,11-Dihydroxy-5β,6β-expoxy-24-methylene-9,11-secocholestan-9-one	*Sinularia granosa*	I	[84]
**321**	6-*epi*-Yonarasterol B	*Echinomuricea* sp.	S,E	[73]
**322**	Carijoside A	*Carijoa* sp.	S,E	[85]

* Inhibition of iNOS (I), COX-2 (C), superoxide anion (S) and elastase (E).

At a concentration of 10 µM, compounds **264**–**275**, **277**–**291**, **293**, **294**, **297**, **303**–**307** and **316**–**320** reduced LPS-induced expression level of iNOS in murine macrophage cells (RAW264.7) [21,74,75,76,77,78,79,80,81,83,84]. Compounds **265**, **266**, **271**, **275**, **277**, **278**, **280**, **281**, **283**–**286**, **288**, **290**, **291**, **293** and **319** suppressed LPS-induced expression level of COX-2 in murine macrophage cells (RAW264.7) [21,74,75,76,77,78,79,80,84]. At 10 µg/mL, compounds **321** and **322** inhibited the generation of superoxide anion and the release of elastase by activated human neutrophils [73,85]. 

### 2.4. Ceramide and Cerebrosides

**Figure 16:**
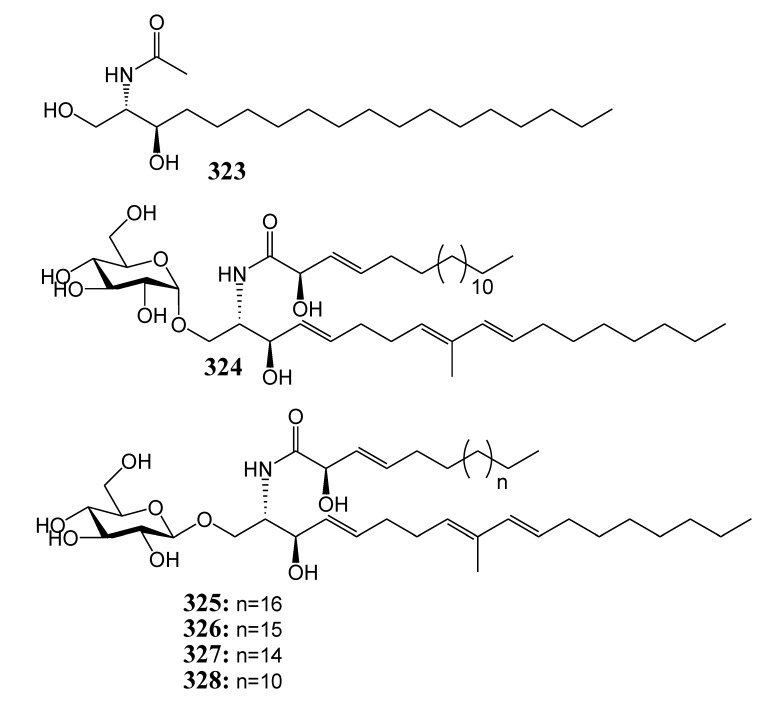
The structures of ceramide and cerebrosides (**323**–**328**).

Table 16 summarizes ceramide (**323**) and five cerebrosides (**324**–**328**) evaluated for *in vitro* anti-inflammatory activity in literature published from 2008 to 2012. The corresponding chemical structures are reported in Figure 16.

**Table 16:** Chemical constituents of ceramide and cerebrosides from soft corals of Taiwan.

No.	Name	Sources	Activities *	Reference
**323**	Ceramide	*Sarcophyton ehrenbergi*	I,C	[86]
**324**	Sarcoehrenoside A	*Sarcophyton ehrenbergi*	I	[86]
**325**	Sarcoehrenoside B	*Sarcophyton ehrenbergi*		[86]
**326**	Cerebroside-3	*Sarcophyton ehrenbergi*	I	[86]
**327**	Cerebroside-5	*Sarcophyton ehrenbergi*	I	[86]
**328**	Cerebroside-6	*Sarcophyton ehrenbergi*	I	[86]

* Inhibition of iNOS (I) and COX-2 (C).

### 2.5. Other Metabolites

**Figure 17:**
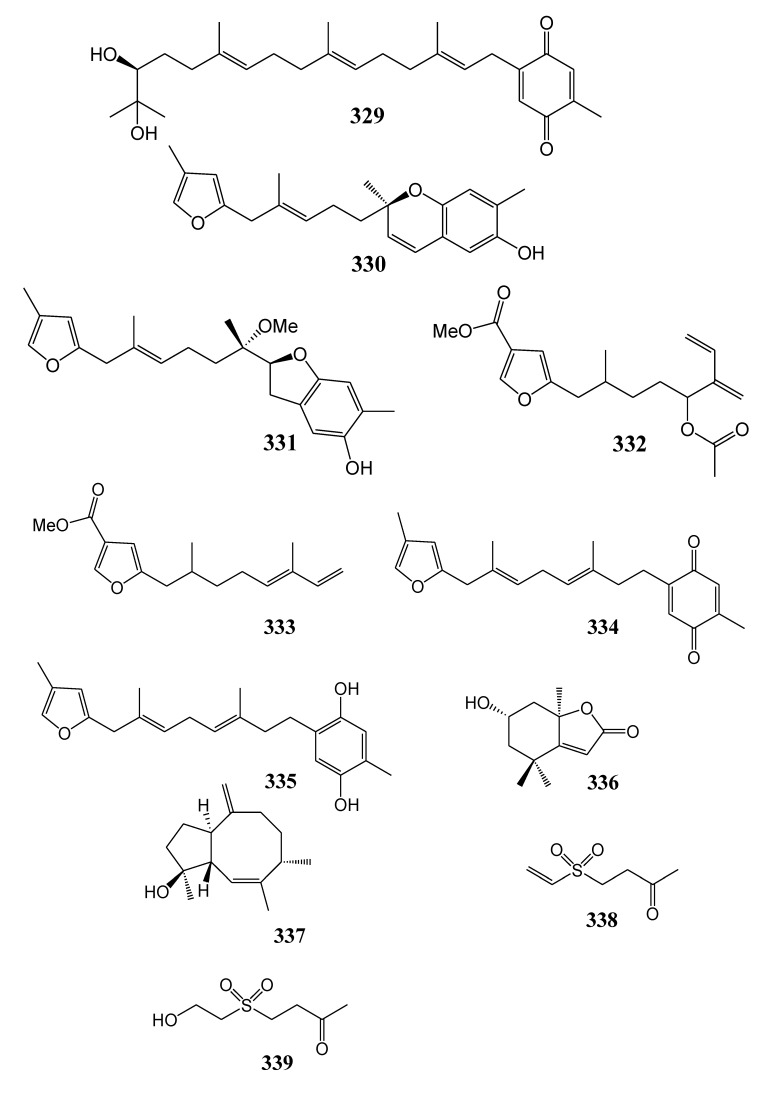
Structures of other metabolites (**329**–**339**).

Table 17 summarizes 11 secondary metabolites of other types (**329**–**339**) evaluated for *in vitro* anti-inflammatory activity in literature published from 2008 to 2012. The corresponding chemical structures are reported in Figure 17.

**Table 17:** Chemical constituents of other metabolites from soft corals of Taiwan.

No.	Name	Sources	Activities *	Reference
**329**	Capilloquinone	*Sinularia capillosa*	I	[87]
**330**	Capillobenzopyranol	*Sinularia capillosa*	I	[87]
**331**	Capillobenzofuranol	*Sinularia capillosa*		[87]
**332**	Capillofuranocarboxylate	*Sinularia capillosa*		[87]
**333**	(*E*)-5-(2,6-Dimethylocta-5,7-dienyl)furan-3-carboxylic acid	*Sinularia capillosa*		[87]
**334**	2-[(2*E*,6*E*)-3,7-Dimethyl-8-(4-methylfuran-2-yl)octa-2,6-dienyl]-5-methylcyclohexa-2,5-diene-1,4-dione	*Sinularia capillosa*	I,C	[87]
**335**	2-[(2*E*,6*E*)-3,7-Dimethyl-8-(4-methylfuran-2-yl)octa-2,6-dienyl]-5-methylbenzene-1,4-diol	*Sinularia capillosa*	I	[87]
**336**	(–)-Loliolide	*Sinularia capillosa*		[87]
**337**	3,4,11-Trimethyl-7-methylenebicyclo[6.3.0]undec-2-en-11R-ol	*Sinularia capillosa*		[87]
**338**	Austrasulfone	*Cladiella australis*		[88]
**339**	Dihydroaustrasulfone alcohol	*Cladiella australis*	I,C	[88]

* Inhibition of iNOS (I) and COX-2 (C).

At a concentration of 10 µM, compounds **323**, **324**, **326**–**330**, **334**, **335** and **339** reduced LPS-induced expression level of iNOS in murine macrophage cells (RAW264.7) [86,87,88]. Compounds **323**, **334** and **339** suppressed LPS-induced expression levels of COX-2 in murine macrophage cells (RAW264.7) [86,88]. Austrasulfone (**338**) was found to exhibit a potent neuroprotective effect in human dopaminergic neuron cells (SH-SY5Y) [89,90]. In animal disease models, the synthetic precursor of austrasulfone dihydroaustrasulfone alcohol (**339**) was not only demonstrated to attenuate neuropathic pain, but also to suppress the progression of multiple sclerosis and atherosclerosis [88].

## 3. Conclusions

Marine invertebrates, particularly octocorals, are rich potential sources of drug leads. Most of our own and other studies on anti-inflammatory activities of natural products from soft corals have been focused on “screening-like” assays using COX-2 and iNOS as target markers. These assay studies have been useful in generating small libraries of anti-oxidant and anti-inflammatory activities from a broad spectrum of soft corals. These results, however, apparently have limitations. For example, the findings are usually generic in nature, and there is often difficulty in immediate or specific application of such results to drug/pharmaceutical discovery, as compared to the existing synthetic chemicals or phytochemicals or those being developed for clinical use. We [45,57,88] and others [25,26] have recently initiated a number of cross-disciplinary studies, employing bio-organic chemistry, cellular immunology and animal disease models for systematic and in-depth studies. As a result, we believe that useful information on the possible application of specific natural products from soft corals for future clinical studies have been obtained. We consider such approaches [57] may need to be encouraged and organized at the international level, and hopefully be integrated into systematic studies, aiming to create translational research of marine natural products for pharmaceuticals/nutraceuticals. Special emphasis may need to be placed on new or specific cell biological/disease model systems.

In terms of evaluating marine natural products for future pharmaceutical application, despite the abundance of unique marine natural products identified, the extremely low quantity of a given compound of interest that can be isolated from marine organisms may be a big hurdle for evaluation of *in vivo* bioactivities and development for pharmaceutical applications.

Fortunately, due to the recent advancement in aquaculture technologies, aquacultural cultivation of various types of specific soft corals is becoming possible. Our team has successfully cultured a number of species of soft corals, including *Klyxum simplex* and *Briareum excavatum* [47,91]. As a result, more abundant and routine preparations of experimental materials will become available for global distribution and collaborative research purposes. Nonetheless, the vast volume of marine organisms and the small base of knowledge so far assembled on soft coral-derived marine chemicals calls for increased international cooperation in this field. 
